# Neuronal nonlinearity explains greater visual spatial resolution for dark **than for **light stimuli

**DOI:** 10.1186/1471-2202-14-S1-P7

**Published:** 2013-07-08

**Authors:** Jens Kremkow, Jianzhong Jin, Stanley J Komban, Yushi Wang, Reza Lashgari, Michael Jansen, Xiaobing Li, Qasim Zaidi, Jose-Manuel Alonso

**Affiliations:** 1 Graduate Center for Vision Research, State University of New York College of Optometry, NY, 10036, USA

## 

**Figure 1:**
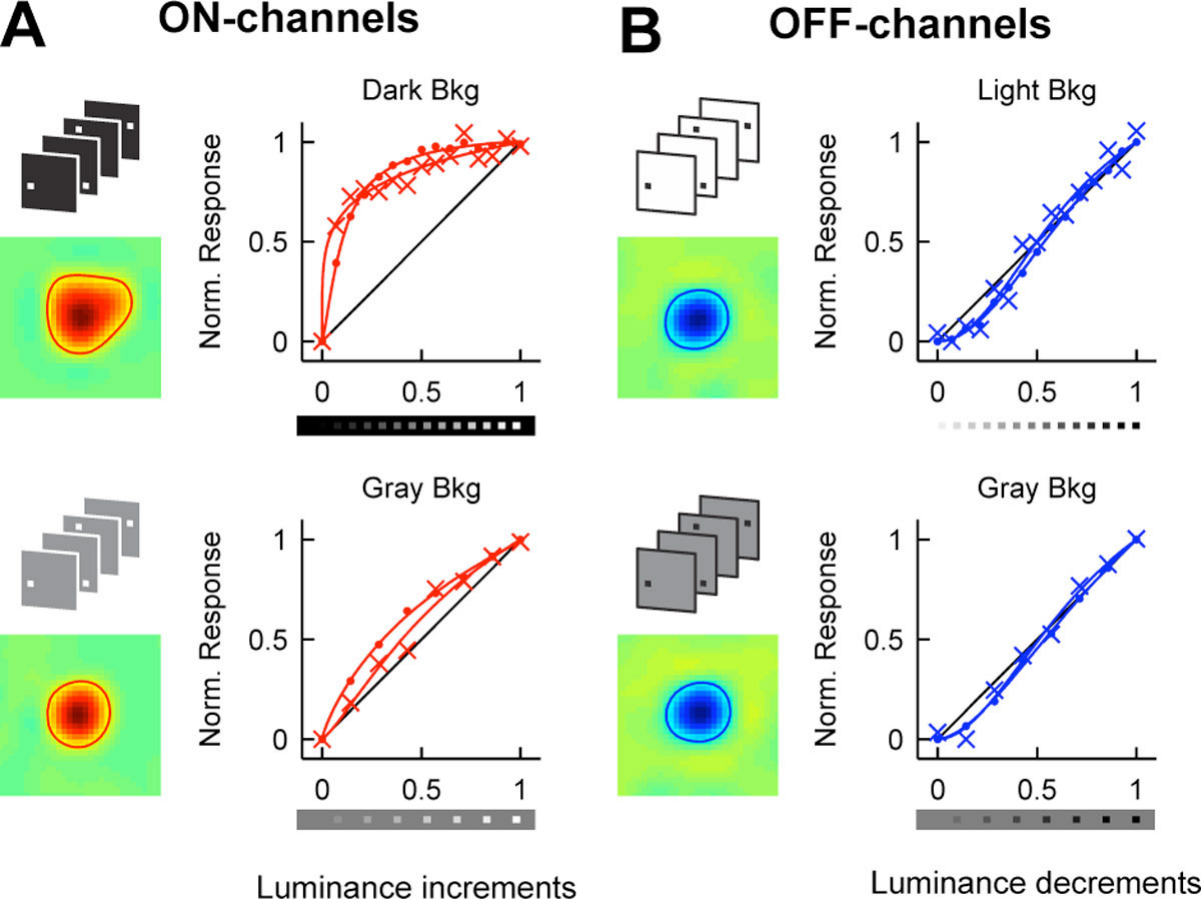
**Functional asymmetries between ON and OFF channels**. ON receptive fields are larger than OFF receptive fields (**A**, **B**; left) on dark but not gray backgrounds. Luminance response functions are more linear in ON (**A**; right) than OFF (**B**; right) channels in the LGN (dots) and V1 (crosses).

Astronomers and physicists noticed centuries ago that visual spatial resolution is higher for dark than light stimuli [1,2], but the neuronal mechanisms for this perceptual asymmetry remain undetermined. We investigated the neuronal mechanisms of this difference in spatial resolution by recording single ON- and OFF-center cells in the visual thalamus (LGN) and multi-unit activity in the visual cortex (V1) of cats. We found that receptive fields of ON-center cells were larger than receptive fields of OFF-center cells in LGN (Figure 1A, B top; ON/OFF = 1.27, p < 0.01) and V1 (ON/OFF = 1.29, p < 0.001), when mapped on binary backgrounds (light targets on dark backgrounds and dark targets on light backgrounds). Surprisingly, when receptive fields were mapped on gray backgrounds, these differences disappeared in the LGN (Figure 1 A, B bottom; ON/OFF = 0.97, p = 0.3) and were slightly reversed in V1 (ON/OFF = 0.8, p = 0.004). Thus, the difference in spatial resolution between ON and OFF neurons is not constant and changes dynamically with the background luminance, as is also the case for the difference in human spatial resolution for darks and lights.

We hypothesized that a nonlinear encoding of luminance increments and decrements could explain these differences. We found that OFF cells in LGN increase their responses roughly linearly (Figure 1B, right) with luminance decrements, independent of the background luminance (luminance half-saturation = L50; LGN OFF L50 light/gray = 0.99, p = 0.9; V1 OFF L50 light/gray = 1.03, p = 0.2). In marked contrast, ON-center cells saturate their responses with small increases in luminance (Figure 1A, top-left) and require bright backgrounds to approach the linearity of the OFF-center cells (Figure 1A, bottom-left; LGN ON L50 dark/gray = 0.28, p < 0.001; V1 ON L50 dark/gray = 0.3, p < 0.001). Although the integration of lights becomes more linear as the luminance of the background increases, ON responses still saturate more than OFF responses on gray backgrounds (V1 L50 darks/lights = 1.25, p < 0.001). Similar differences in response linearity could be demonstrated in recordings from local field potentials in awake primates.

Consistent with our hypothesis, we show that a simple computational model based on the ON-channel nonlinearity can explain the larger receptive fields and lower spatial resolution of ON cells, the differences in human spatial resolution between darks than lights, the differences in the size of ON and OFF dendritic fields from retinal ganglion cells and the recently demonstrated OFF dominance in visual cortex (larger number of OFF- than ON-dominated cortical neurons). These results demonstrate a fundamental difference in processing between ON and OFF channels, which could have major implications in visual perception and cortical development.
